# Bile acids induce liver fibrosis through the NLRP3 inflammasome pathway and the mechanism of FXR inhibition of NLRP3 activation

**DOI:** 10.1007/s12072-023-10610-0

**Published:** 2024-01-03

**Authors:** Shu Feng, Xingming Xie, Jianchao Li, Xu Xu, Chaochun Chen, Gaoliang Zou, Guoyuan Lin, Tao Huang, Ruihan Hu, Tao Ran, Lu Han, Qingxiu Zhang, Yuanqingxiao Li, Xueke Zhao

**Affiliations:** 1 Department of Infectious Diseases, Affiliated Hospital of Guizhou Medical University, Guizhou Medical University, No. 9 Beijing Road, Guiyang, 550004 Guizhou China; 2 Department of Hepatobiliary Surgery, The First Affiliated Hospital of Guangzhou Medical University, Guangzhou, 510120 Guangdong Province China; 3 Laboratory of Hepatology, Affiliated Hospital of Guizhou Medical University, Guizhou Medical University, Guiyang, 550004 Guizhou China; 4 Department of Hepatobiliary Surgery, Affiliated Hospital of Guizhou Medical University, Guizhou Medical University, Guiyang, 550004 Guizhou China; 5 Department of Cardiovascular Medicine, Guiqian International General Hospital, Guiyang, 550018 Guizhou China

**Keywords:** Bile acids, Farnesoid X receptor, NLRP3, GCDCA, Hepatic fibrosis

## Abstract

**Background:**

Altered patterns of bile acids (BAs) are frequently present in liver fibrosis, and BAs function as signaling molecules to initiate inflammatory responses. Therefore, this study was conducted to uncover the notably altered components of BAs and to explore the pathway of altered BA induced inflammation in the development of liver fibrosis.

**Methods:**

Bile acids were quantified by ultraperformance liquid chromatography coupled to mass spectrometry (UPLC‒MS/MS). Cell Counting Kit-8 assays were used to determine the proliferative capacity of HSCs. Transwell assays and wound healing assays were used to determine the migratory capacity of LX2 cells. Protein expression was evaluated by western blotting.

**Results:**

Plasma bile acid analysis showed higher levels of GCDCA, TCDCA, GCA and TCA in patients with liver fibrosis than in normal controls. The AUC of GCDCA was the highest. Western blotting showed that GCDCA treatment increased the expression of NLRP3-related proteins and collagen1 in vitro and significantly increased LX2 cells proliferation and migration. Furthermore, knockdown of NLRP3 or overexpression of FXR in LX2 cells decreased the expression of the above proteins, and FXR inhibited NLRP3 (ser 295) phosphorylation in vitro and vivo. In vivo, HE, Masson’s trichrome, and Sirius Red staining showed that GCDCA increased collagen fibers in the mouse liver, and the expression of NLRP3-related proteins, collagen 1, and α-SMA in the liver increased significantly. However, the knockout of NLRP3 reversed these patterns.

**Conclusion:**

(1) Primary conjugated bile acids increased in patients with liver fibrosis; (2) GCDCA induce hepatic fibrosis via the NLRP3 inflammasome pathway; (3) FXR inhibits NLRP3 activity by restraining its phosphorylation; (4) knockdown or knockout of NLRP3 may relieve the onset of hepatic fibrosis.

**Supplementary Information:**

The online version contains supplementary material available at 10.1007/s12072-023-10610-0.

## Introduction

Hepatic stellate cells (HSCs) play a pivotal role in the development of liver fibrosis, and activated HSCs produce extracellular matrix (ECM), mainly composed of collagen. Excessive ECM is deposited in the portal area of the liver to form collagen fibers, which destroys the normal structure and function of the liver and stimulates inflammatory signaling pathways [1]. Chronic liver disease, caused by virus, alcohol, fat, drug, and autoimmunity, will result in the progression of fibrosis. Along with the progress of the disease and the constant stimulation of inflammation, cirrhosis and even hepatocellular carcinoma will occur [1]. Therefore, it is especially important to understand the pathogenesis of inflammation during hepatic fibrosis.

Under physiologic conditions, primary BAs are metabolized by cholesterol in the liver and then enter the intestine through the bile duct. In this process, BAs in hepatocytes are mainly transferred to the bile duct through various transporters, such as bile salt export pumps (BSEP), MRP2 and MDR3 [2]. After the primary conjugated bile acids enter the intestine from the liver, they are metabolized into secondary bile acids under the action of bacteria [3]. However, when the intrahepatic bile ducts are compressed by the fibrotic liver tissue, bile acids produced in the liver are unable to enter the intestine via the bile duct completely, so the accumulated bile acids will be transported to the systemic circulation through transporters MRP3, MRP4 and OST α/β on the lateral basement membrane of hepatocytes, which are rarely expressed under normal physiological conditions [2]. A study showed that with the development of liver fibrosis, the composition of bile acids in liver, bile and plasma had changed, and bile acids produced by hepatocytes were discharged into peripheral plasma [4]. Therefore, the bile acid spectrum in plasma may reflect the metabolism of bile acid in the liver to some extent [5]. In a systematic review and meta‑analysis, BAs included glycocholic acid (GCA), glycochenodeoxycholate (GCDCA), taurocholic acid (TCA), and taurochenodeoxycholate (TCDCA), which may increase with the development of cirrhosis [6]. In nonalcoholic steatohepatitis (NASH), the total serum and fecal BA levels generally increased, and the increase of total serum BAs was positively correlated with the severity of the disease [7]. However, BAs changes in plasma during liver fibrosis are not fully understood.

NACHT, LRR, and PYD domain–containing protein 3 (NLRP3), the most understood Nod like receptors (NLRs), can be activated by damage-associated molecular patterns (DAMPS) and pathogen-associated molecular patterns (PAMPS) [8]. Under stimulation, the NLRP3 inflammasome complex was assembled by NLRP3, apoptosis-associated speck-like protein containing a CARD (ASC) and pro-caspase-1, which leads to activation of caspase-1, which subsequently triggers the cleavage of pro-IL-1β into its mature pro-inflammatory cytokine IL-1β [8, 9]. BAs can activate NLRP3 inflammasome as signal 1 or signal 2 [10, 11]. In mouse experiments, BA (DCA or LCA) feeding can cause liver damage and activation of NLRP3 inflammasome. In vitro, DCA can upregulate the expression of NLRP3 and IL-1β, and increase the activity of the effector molecule Caspase-1. While these inflammatory signals and fibrotic phenotype were ameliorated after NLRP3 knockout in either mice or cells [10]. However, there has been no research on the effects of bile acids on the proliferation and migration of HSCs, which play a key role in the process of liver fibrosis. Furthermore, studies showed that various receptors, such as farnesoid X receptor (FXR) and Takeda G-protein receptor 5 (TGR5), can be regulated by BAs, which are important to inflammatory reactions, the liver-gut axis, bacterial growth, lipid, glucose and amino acid metabolism [6, 12–14]. FXR, as a nuclear receptor, can be activated by certain endogenous BAs ligands (CDCA > DCA > LCA > CA) [13, 14]. In the hepatocyte, cytochrome p450 CYP7A1 is the rate-limiting enzyme in the process of cholesterol metabolism to BAs. While FXR can inhibit the expression of CYP7A1 through the downstream molecule small heterodimer partner (SHP), thus inhibiting the production of BAs [7, 14]. Moreover, BAs, proven to be DAMPs, can activate NLRP3 inflammasome during cholestasis-associated sepsis and FXR plays pivotal roles in sepsis and endoplasmic reticulum (ER) stress by controlling the NLRP3 inflammasome [11, 15]. However, the mechanisms underlying this protection are not completely elucidated.

In this work, we investigated the role of BAs in liver fibrogenesis and the mechanism of FXR-mediated negative regulation of NLRP3 inflammasome activation. Therefore, we could discover new targets for the prevention, diagnosis, and treatment of chronic liver diseases, especially liver fibrosis.

## Materials and methods

### Hepatic fibrosis patients and healthy individuals

Plasma samples were collected from patients with biopsy proven-fibrosis (n = 75) from Infection Department in the Affiliated Hospital of Guizhou Medical University (Guiyang, China). Healthy control group inclusion criteria were as follows: (1) Age over 18 years old. (2) Normal hepatic functions, such as alanine transaminase (ALT) and aspartate transaminase (AST) within the normal range, were present in the liver. (3) Abdominal ultrasonography showed no obvious abnormalities in the liver and gallbladder. (4) In the absence of infection with hepatitis B or C viruses.

Twenty-five normal control plasma samples were collected from the Physical Examination Center in the Affiliated Hospital of Guizhou Medical University. The pathological diagnosis of liver tissue refers to the classification and staging standard of chronic hepatitis in the Prevention and Treatment Plan of Viral Hepatitis issued in 2000 in China [16]. To be brief, S0 was defined as no fibrosis in the liver specimens; S1 was defined as portal fibrosis enlarged, and localized fibrosis around sinuses and lobules; S2 was defined as fibrosis around portal area, formation of fibrous septa and retention of lobular structure; S3 was defined as fibrous septal defects with lobular structural disorder and absence of cirrhosis, and S4 was defined as early cirrhosis [16].

Fibrous liver tissue and control samples

Liver specimens from 15 patients with liver fibrosis were collected from the Department of Infectious Diseases, Affiliated Hospital of Guizhou Medical University between March 2021 and September 2021. Samples diagnosed as S0 (no fibrosis in the liver specimens) by pathological diagnosis after liver diagnostic puncture in Infectious Diseases Department were used as normal controls (n = 15). None of the mentioned subjects had contraindications to liver biopsy, and the study was approved by the Ethics Committee of the Affiliated Hospital of Guizhou Medical University (Approval 2020, Ethics Review No. 205). All participants signed informed consent for the study.

### Quantification of BAs

The BAs concentration in the plasma samples was quantified using ultraperformance liquid chromatography coupled with triple quadrupole mass spectrometry (UPLC-TQMS, Waters, Milford, MA), accomplished by Health Bank Medical Inspection Office Co., Ltd (Hangzhou, China). BAs detected in plasma include CA, CDCA, GCDCA, GCA, TCA, TCDCA, LCA, GUDCA, TUDCA, UDCA, GDCA, TDCA, DCA, GLCA and TLCA.

### Cell lines

LX-2 human HSCs were purchased from Zhongqiao Xinzhou (Shanghai, China) authenticated by STR, and had no mycoplasma contamination. All cell lines were cultured in DMEM (GibcoBRL, Rockville, MD, USA) with 10% fetal bovine serum (04–001-1A, Biological Industries) and incubated at 37 °C with 5% CO2.

### Cell treatment

LX2 cells were primed with 500 ng/ml LPS for 6 h before stimulation with GCDCA at different concentrations for the same time or at the same concentration for different times [18]. Then, supernatants were harvested at the indicated time points, and the IL-1β level was determined by a human IL-1β ELISA kit purchased from Jiangsu Meimian Industrial Co., Ltd. (Jiangsu, China), according to the manufacturer’s instructions.

### NLRP3 knockdown and FXR overexpression lentivirus infection

ShRNA knockdown lentiviral particles for human NLRP3 and their controls were synthesized by GeneChem (Shanghai, China). Overexpression lentiviral particles for human FXR (OE-FXR) and their controls were purchased from Hanheng (Shanghai, China). All infections were performed according to the manufacturer’s instructions.

### Mice

C57BL/6 J male mice (aged 7 weeks, weighing 20 ± 3 g) were purchased from the Beijing Vital River Laboratory Animal Technology Co., Ltd. (Beijing, China), and NLRP3-deficient mice were purchased from Shanghai Model Organisms Center, Inc., (Shanghai, China). The method of constructing NLRP3 knockout homozygous mice is briefly described as follows: In this study, CRISPR/Cas9 technology is used to introduce mutations using non-homologous end joining, resulting in transcoding of the NLRP3 gene's reading box protein and functional deletion. Briefly, Cas9 mRNA and gRNA were obtained by in vitro transcription. To obtain F0 generation mice, Cas9 mRNA and gRNA were microinjected into the zygotes of the C57BL/6 J mouse. PCR amplification and sequencing identified F0 positive mice mated with C57BL/6 J mice to yield F1 positive mice (NLRP3^+/−^). Heterozygous mice were self-crossed to obtain gene-knockout homozygous mice (NLRP3^−/−^).

The mice were kept in SPF animal house and adapted for one week before intervention. The animal study was approved by the Animal Ethics Committee of the Hospital Affiliated with Guizhou Medical University (NO.2000732).

### Liver fibrosis mouse models

Model 1: The role of NLRP3 in Carbon tetrachloride (CCl_4_)-induced liver fibrosis in C57BL/6 J mice.

Eight wild-type (WT) male C57BL/6 J mice were randomly divided into three groups, and 8 NLRP3 knockout male mice were randomly divided into two groups (n = 4 in each group): (1) the vehicle group (intraperitoneal injection (ip) with corn oil as vehicle); (2) the CCl_4_ group (ip with 20% CCl_**4**_ – corn oil solution at 5 μl/g body weight); (3) the NLRP3^−/−^ + oil group (ip with corn oil); and (4) the NLRP3^−/−^ + CCl_4_ group (ip with 20% CCl_4_–corn oil solution at 5 μl/g body weight). They were all three times a week for 12 weeks.

Model 2: To verify the role of NLRP3 and FXR in GCDCA-induced hepatic fibrosis in C57BL/6 J mice respectively.

The experiment was divided into four groups (n = 5 in each group), namely (1) the control group (10% DMSO + 40% PEG300 + 5% Tween-80 + 45% saline); (2) the GCDCA gavage group: GCDCA powder was dissolved in 10% DMSO, 40% PEG300, 5% Tween-80 and 45% saline in turn. The mice were fed GCDCA at a dose of 1000 mg/kg body weight using a specific gavage needle; (3) the GW4064 (30 mg/kg body weight) + GCDCA (1000 mg/kg body weight) gavage group: the dissolution of GW4064 was the same as that of GCDCA and (4) the NLRP3^−/−^ mice with GCDCA (1000 mg/kg body weight) gavage group. All mice were given about 200ul solution each time. They were all three times a week for 12 weeks.

All mice were sacrificed 24 h after the final challenge and all operations followed the principle of sterility.

### Western blot analysis

Western blot analysis was performed as previously described [17]. Primary antibodies included anti-GAPDH (1:7000, 10494–1-AP, Proteintech, China), anti-β-actin (1:8000, P30002, Abmart), anti-FXR (1:1000, 25055–1-AP, Proteintech, China), anti-caspase-1 (1:1000, 22915–1-AP, Proteintech, China), anti-IL-1β (1:1000, 16806–1-AP, Proteintech, China), anti-SMA (1:1000, 14395–1-AP, Proteintech, China), anti-ASC (1:500, 10500–1-AP, Proteintech, China), anti-collagen I (1:1000, ab260043, Abcam), anti-phospho-NLRP3 (Ser 295) (1:500, TA4320, Abmart) and anti-NLRP3 (1:1000, ab263899, Abcam). The secondary antibodies included HRP-conjugated Affinipure goat anti-rabbit IgG (H + L) (1:7000, SA00001-2, Proteintech, China) and HRP-conjugated Affinipure goat anti-mouse IgG (H + L) (1:7000, SA00001-1, Proteintech, China).

### Cell proliferation assays

Cell Counting Kit-8 (CCK-8, Dojindo) assays were utilized for cell proliferation analysis according to the manufacturer’s instructions. In brief, 10^4^ cells (100 μL/well) were seeded in 96-well plates with 100 µl complete medium (control), 100 µl LPS (500 ng/ml), or 100 µl LPS (500 ng/ml) + GCDCA (100 μΜ). LX2 cells were primed with 500 ng/ml LPS for 6 h before stimulation with GCDCA. After the culture plate was placed in an incubator for preincubation (37 °C, 5% CO2), 10 μL of CCK-8 solution was added to each well for 2 h, and then, the culture plate was evaluated with a microplate reader to detect the OD value at 450 nm.

### Cell migration assays

Wound-healing and transwell assays were used to evaluate migration. LX2 cells were inoculated into 6-well plate, and when they grew to 100% confluence, the cell monolayer was wrapped with a 200 μL pipette tip. The wound was photographed, which served as the 0-h time point. Cells were incubated in serum-free culture medium for 48 h, and wound healing was photographed. For the transwell assay, LX2 cells (2 × 10^4^ cells/200 µL) were seeded in the top chamber with 5% serum in a 24-well polycarbonate transwell filter (8 µm pore size, Corning Incorporated, USA), which was filled with 20% fetal bovine serum (700 µL) and placed in the lower chamber. After 48 h of incubation, cells grown in the polycarbonate transwell filter were fixed with 4% paraformaldehyde and stained with 0.1% crystal violet. The cells were wiped from the apical chamber with a cotton swab, and migrated cells were photographed with an inverted microscope [17]. The grouping and processing of cells are the same as CCK8 assay.

### Chromatin immunoprecipitation (ChIP) assay

We conducted a ChIP assay in Flag-FXR-overexpressing LX2 cells using a simple ChIP enzymatic chromatin IP kit (Cell Signaling) according to the manufacturer’s protocol. qPCR was utilized to verify the presence of FXR-binding region in the NLRP3 promoter. The following qPCR primer sequences were used: (1) forward, 5ʹ-TGGGATTACAGGCGTGAG − 3ʹ and reverse, 5ʹ-CTGGGTGACAAGAGCAAGAC − 3ʹ; (2) forward, 5ʹ-TGAGTCAATGAGTCAGGGAG − 3ʹ and reverse, 5ʹ- GAGGGAAGTGAAACTAAGGA − 3ʹ. The antibodies included anti-normal rabbit IgG (Cell Signaling Technology, #2729) and anti-Flag (Cell Signaling Technology, #14793).

### Dual-luciferase reporter assay

The effect of FXR on the NLRP3 promoter was determined by cotransfecting pcDNA FXR or pcDNA-vector (NC) into 293 T cells with PGL3-based constructs containing an empty sequence WT or MT1/MT2 NLRP3 promoter sequence, and Renilla luciferase reporter plasmids. Twenty-four hours after transfection, the luciferase activity of firefly and Renilla was measured with a luciferase reporter assay kit (Genomeditech, Shanghai, China). The ratio of firefly luciferase activity to Renilla luciferase activity was calculated for each sample.

### Coimmunoprecipitation (COIP)

LX2 cells overexpressing Flag-FXR were harvested and protein samples were extracted. Samples of 100ul, 100ul, 300ul, 300ul were taken into four EP tubes, identified as Input, IgG, IP-Flag and IP-NLRP3 groups. Add 5 × loading buffer 25ul to the input group, boil for 10 min, storage at -20 °C. Protein A / G Plus Agarose was added to the IgG and IP groups at 10ul, respectively, and 4℃ rotated for 1 h, then 4℃, 12,000 rpm centrifuge for 10 min, supernatant taken. Anti-IgG antibody was added to the IgG group, anti-Flag antibody (1:50) and anti-NLRP3 antibody (1:200) were added to the IP groups, rotated in 4℃ for 3.5 h, then 40ul protein A/G Plus-Agarose were added, 4℃ rotated overnight, then 4℃, 3500 rpm centrifuged for 10 min, retained sediment, 120ul 1 × loading buffer added, mixed, and boiled at 100℃. And then immunoblotting was followed. The reagents mainly included anti-NLRP3 (Cell Signaling Technology, #15101), anti-flag (Cell Signaling Technology, #14793), anti-IgG (Abmart, B30011) and protein A/G Plus-Agarose (Santa Cruz, sc-2003).

### Hematoxylin & eosin (H&E), Masson’s trichrome, and sirius red staining

H&E staining kits (G1120) and Masson’s Trichrome Stain Kit (G1340) were purchased from Solarbio Biotechnology Co., Ltd. (Beijing, China), and Sirius Red staining solution was purchased from Siweiga biotech Co., Ltd (Wuhan, China). They were used according to the manufacturer’s guidelines.

### Immunohistochemistry

Briefly, paraffin sections were subjected to conventional dewaxing in water, antigen retrieval, endogenous peroxidase blocking, serum blocking, primary antibody incubation, secondary antibody incubation, DAB chromogen, counterstained nuclei, dehydrated slides, Microscopic examination, and finally Image acquisition analysis were performed.

Primary antibodies included anti-α-SMA (1:200, bs-10196R, Bioss, China), anti-NLRP3 (1:200, 19771–1-AP, Proteintech, China), anti-phospho-NLRP3 (ser 295) (1:200, TA4320, Abmart, China) and anti-IL-1β (1:200, bs-0812R, Bioss, China).

### Chemicals and reagents

GW4064, PEG300, Tween 80 and GCDCA were purchased from MedChemExpress. LPS (L2880) was purchased from Sigma. CCl_4_ was purchased from Lianlong Bohua (Tianjin) Pharmaceutical Chemistry Co., Ltd. (Tianjin, China).

### Statistical analysis

Student’s *t* test or Mann-Whitney U test was used for two groups. GraphPad Prism 9 (GraphPad Software, USA) was utilized for statistical analysis and to generate graphs. Diagnosis and prediction analyses were performed using receiver operating characteristic curves. p < 0.05 was considered statistically significant.

## Results

### Alterations in the plasma levels of bile acids and inflammation in patients with liver fibrosis

**Fig. 1:**
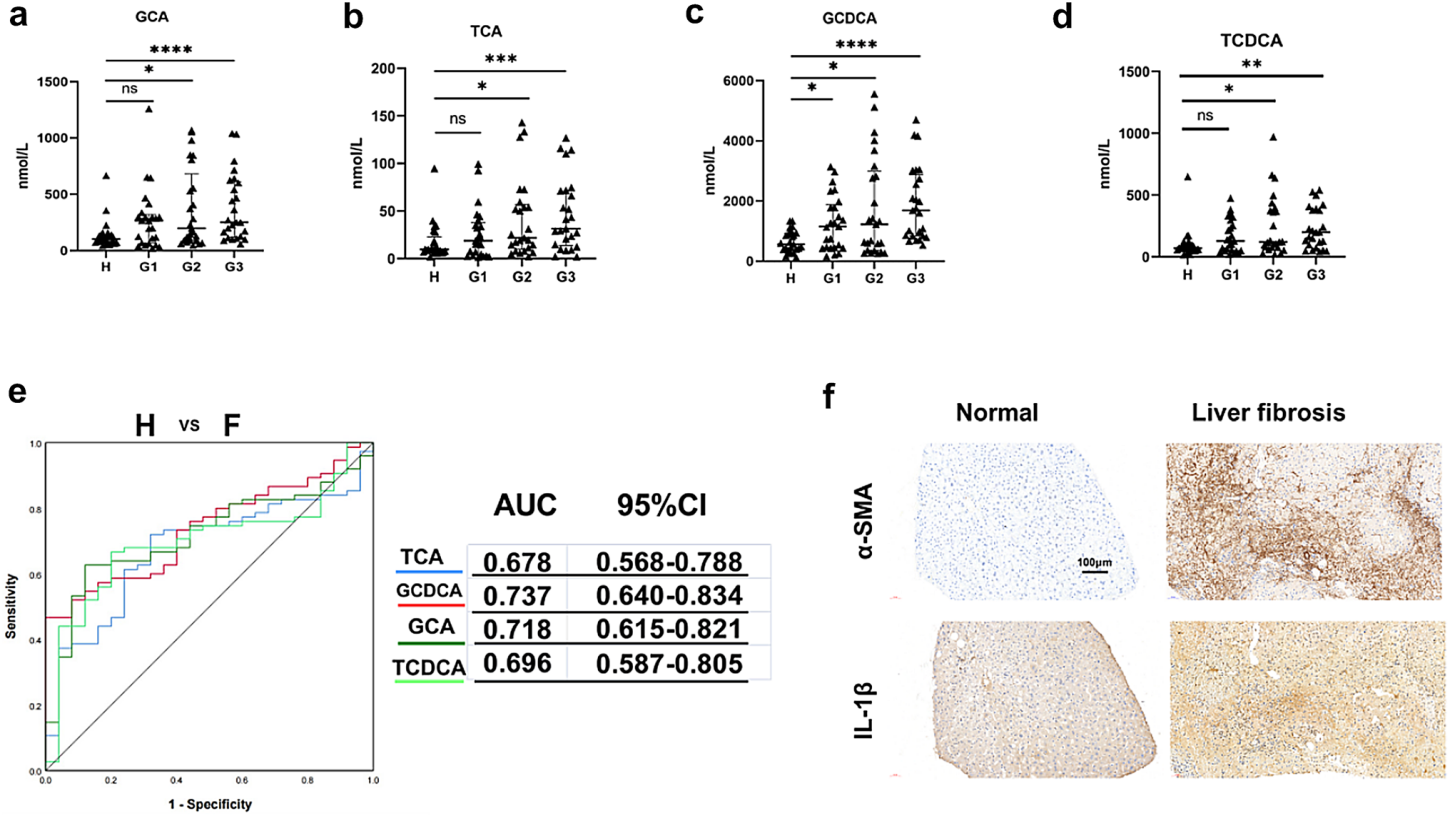
Alterations in bile acids in plasma and inflammation in patients with liver fibrosis. The concentrations of GCA **(a)**, TCA **(b)** GCDCA **(c)** and TCDCA **(d)**. Mann–Whitney U test were used. *p < 0.05; **p < 0.01; ***p < 0.001; ****p < 0.0001. **e** ROC curve analysis was performed for H (healthy individuals) and F (fibrosis patients). **f** Immunohistochemistry of IL-1β and α-SMA between normal and fibrosis livers. Scale bar: 100 μm

A total of 75 patients with biopsy proven-fibrosis were included in this study and all had viral hepatitis as a cause, of which 70 were hepatitis B virus cases and 5 hepatitis C virus cases. We defined 25 patients with stage 1 fibrosis as G1 group, 25 patients with stage 2 fibrosis as G2 group, and 25 patients with stage 3 and 4 fibrosis as G3 group. Twenty-five healthy individuals as a control group were defined as group H. In this study, we measured the concentrations of 15 BAs in plasma samples collected from all participants. Among the four groups, four conjugated primary BAs (GCA, TCA, TCDCA and GCDCA) were able to discriminate between the liver fibrosis group and the group of healthy subjects (Fig. 1a–d). Other BAs had no significant statistical differences (Fig. S1). To examine the diagnostic values of these four significantly altered bile acids, we performed receiver operating characteristic (ROC) analysis (Fig. 1e, left). The area under the curve (AUC) value of GCDCA between hepatic fibrosis patients and healthy individuals was the largest (Fig. 1e, right). The patients with liver fibrosis had significantly higher positivity for α-SMA and IL-1β by immunohistochemistry than normal subjects (Fig. 1f). Of the individual BAs, GCDCA has the potential diagnostic value for liver fibrosis, and we therefore chose it as a follow-up intervention.

### GCDCA promotes the activation of LX2 by upregulating the expression of the NLRP3 inflammasome

**Fig. 2:**
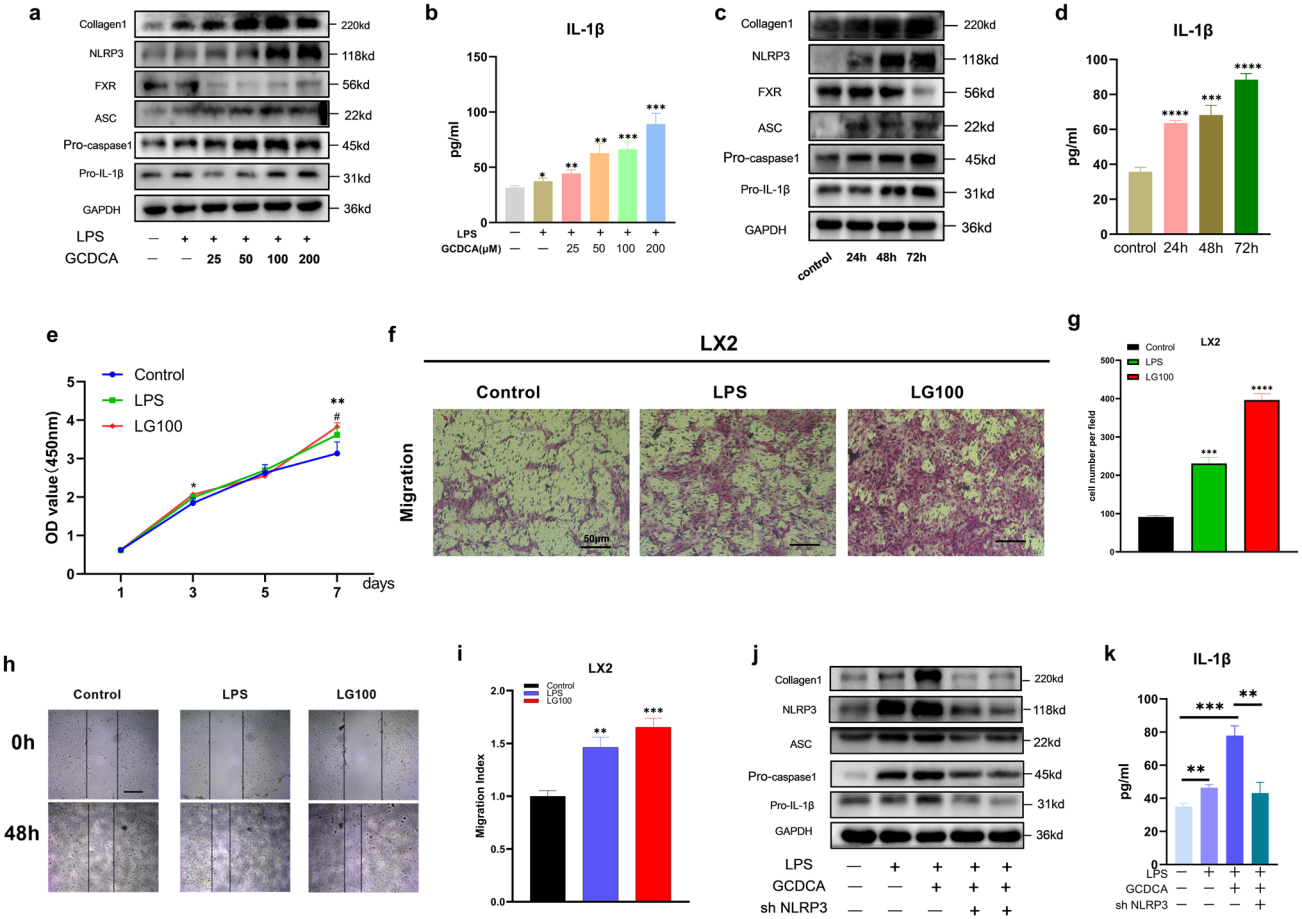
GCDCA activates LX2 through the NLRP3 inflammasome. **a**, **b** LPS-primed LX2 cells were incubated with various doses of GCDCA (μM) for 48 h. **c**, **d** LPS-primed LX2 cells were treated with GCDCA (100 μM) for different time periods. **a**, **c** The protein expressions of NLRP3, pro-IL-1β, collagen I, FXR, pro-caspase-1 and ASC by western blots. **b**, **d** Secreted IL-1β was analyzed by ELISA. **e** CCK-8 assay of LPS-primed LX2 cells treated with GCDCA (100 μM). *LG100 compared with control, #LPS compared with control (p < 0.05). **f** Transwell assay of LX2 cells (scale bar: 50 μm). **g** The number of cells migrating in different groups. **h**, **i** The migratory property of LX2 cells was assessed with a wound healing assay (scale bar: 400 μm). **j**, **k** LPS-primed LX2 cells were treated with GCDCA (100 μM) for 48 h with or without NLRP3 knockdown. **j** Western blot analysis of NLRP3, pro-IL-1β, collagen I, pro-caspase-1 and ASC protein expressions in LX2 cells. **k** Secreted IL-1β was analyzed by ELISA. *p < 0.05; **p < 0.01; ***p < 0.001; ****p < 0.0001. Error bars indicate SEM. Date are from at least 3 independent experiments. LG100: LPS 500 ng/ml + GCDCA 100 μM

GCDCA exposure clearly induced NLRP3 inflammasome-related protein expression (Fig. 2a, c) and IL-1β secretion in a dose- and time-dependent manner (Fig. 2b, d). Liver fibrosis-related protein (collagen 1) expression was also increased with increasing GCDCA concentration and intervention time (Fig. 2a, c). After incubation with GCDCA for 7 days, LPS-primed LX2 cells exhibited a significant increase in proliferation compared to the control cells (Fig. 2e). In addition, the migration of LX2 cells was enhanced after administration of LPS combined with GCDCA (Fig. 2f–i). Moreover, pretreatment with NLRP3 knockdown reversed the upregulation of proteins associated with the NLRP3 inflammasome and liver fibrosis induced by GCDCA in LPS-primed LX2 cells, and IL-1β secretion was also reduced (Fig. 2j, k). Collectively, these data indicate that GCDCA induces the activation of LX2 by increasing the expression of the NLRP3 inflammasome.

### ***NLRP3 knockout reduces CCl***_***4***_***-induced liver fibrosis in mice***

**Fig. 3:**
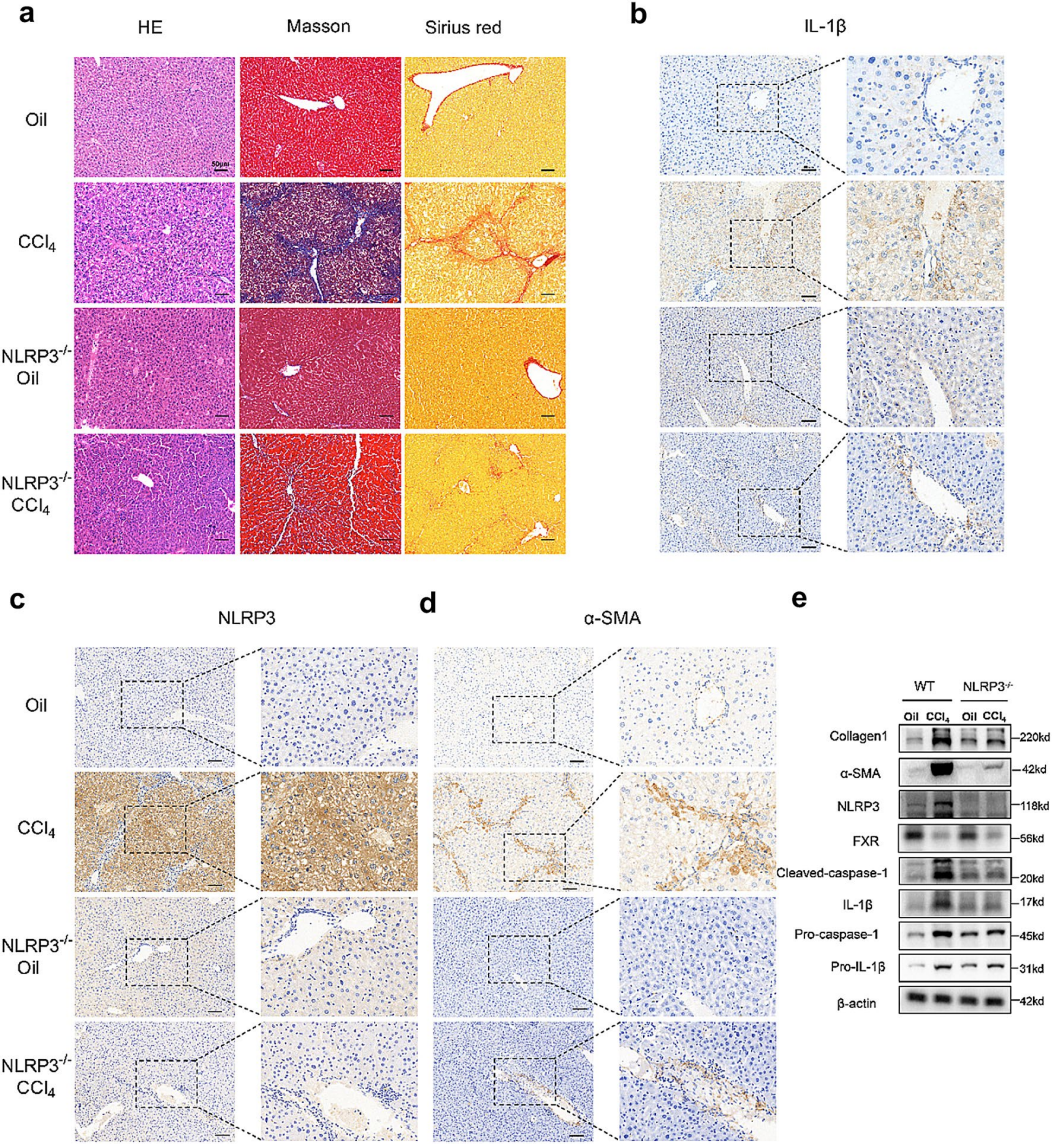
NLRP3 knockout reduces CCl_4_-induced liver fibrosis in mice. **a** HE, Masson and Sirius red staining of mouse livers. Scale bar: 50 μm. **b**, **c**, **d** Immunohistochemistry of IL-1β, NLRP3 and α-SMA in mouse livers. Scale bar: 50 μm. **e** Western blots of NLRP3-related proteins and fibro-related proteins in WT groups and NLRP3^−/−^ groups

To test the role of NLRP3 in liver fibrosis, we used NLRP3 knockout (NLRP3^−/−^) mice and intraperitoneally injected CCl_4_, which has been proved to cause liver fibrosis [18]. By performing HE, Masson’s trichrome, and Sirius Red staining, we found a significant liver fibrosis and collagen formation in CCl4 mice than in oil mice, while NLRP3^−/−^ mice after CCl_4_ intervention had significantly weaker fibrosis than the mice in the CCl_4_ group (Fig. 3a). Immunohistochemistry of the liver showed that the positivity of α-SMA, NLRP3, and IL-1β was significantly higher in the CCl_4_ group but reversed in the NLRP3^−/−^ + CCl_4_ group (Fig. 3b–d). In addition, western blot analysis of the liver showed higher expression of NLRP3, pro-IL-1β, pro-caspase-1, cleaved caspase-1, mature IL-1β (17kd) and fibrosis markers (collagen I and α-SMA) in the CCl_4_ group than in the oil group, while NLRP3 knockout changed the patterns of increase (Fig. 3e).

### NLRP3 knockout reduces GCDCA-induced hepatic inflammation and fibrosis in mice

**Fig. 4:**
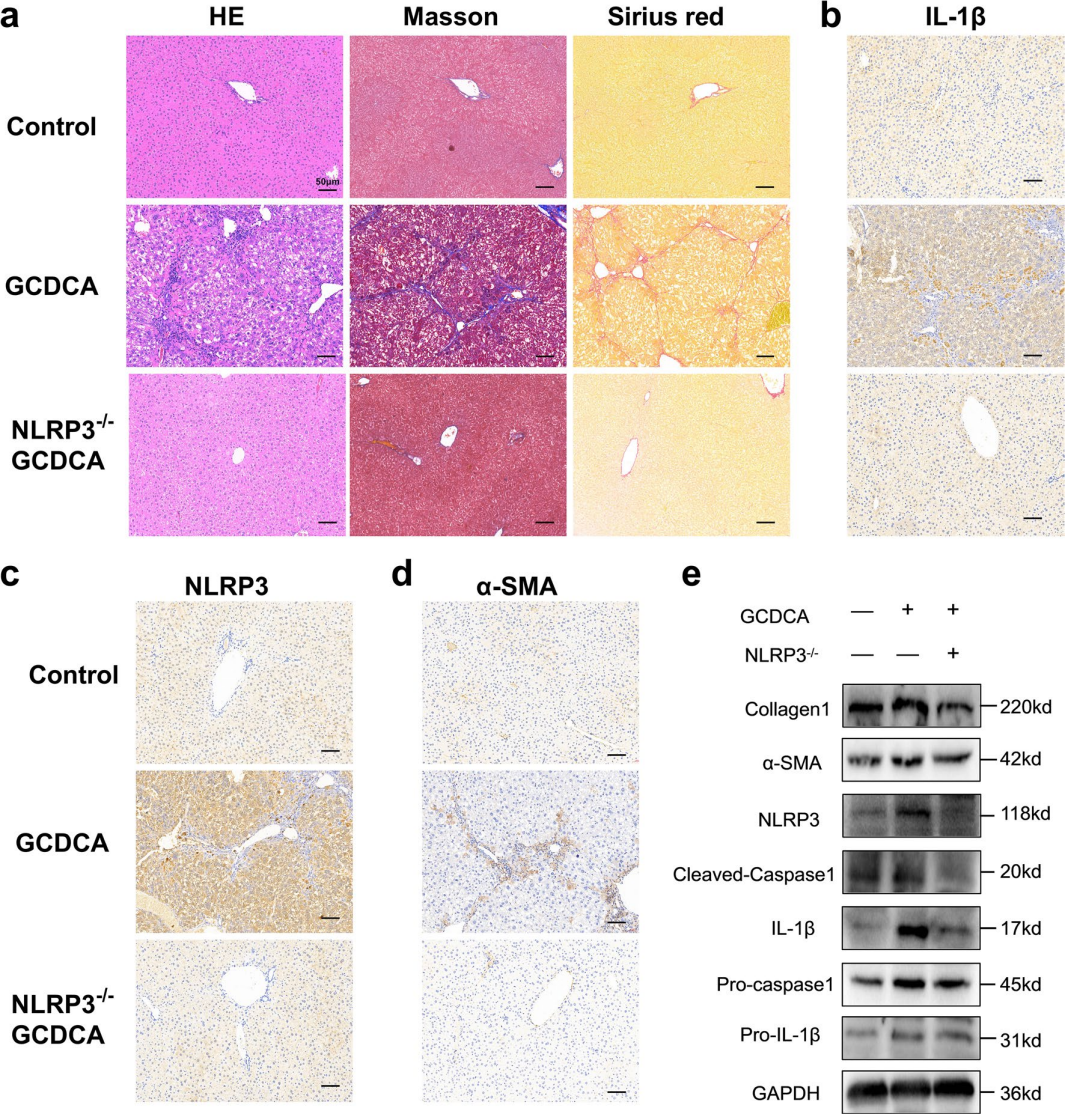
NLRP3 knockout reduces GCDCA-induced liver fibrosis in mice. **a** HE, Masson and Sirius red staining of mouse livers. Scale bar: 50 μm. **b**, **c**, **d** Immunohistochemistry of IL-1β, NLRP3 and α-SMA in mouse livers. Scale bar: 50 μm. **e** Western blots of NLRP3-related proteins and fibro-related proteins among groups

In this study, we performed GCDCA gavage to test whether BA could induce liver fibrosis in mice. HE, Masson’s trichrome, and Sirius Red staining showed that GCDCA increased fibrosis and collagen formation in mouse livers (Fig. 4a). In addition, immunohistochemistry of the liver showed significantly higher positivity of α-SMA, NLRP3, and IL-1β in the GCDCA group than in the control group (Fig. 4b–d). The protein expressions of NLRP3 inflammasome and fibrosis markers in the GCDCA group were also higher than the control group (Fig. 4e). However, the knockout of NLRP3 could reduce the occurrence of liver inflammation and fibrosis in mice (Fig. 4).

### FXR attenuates liver fibrosis by inhibiting the phosphorylation of NLRP3

**Fig. 5:**
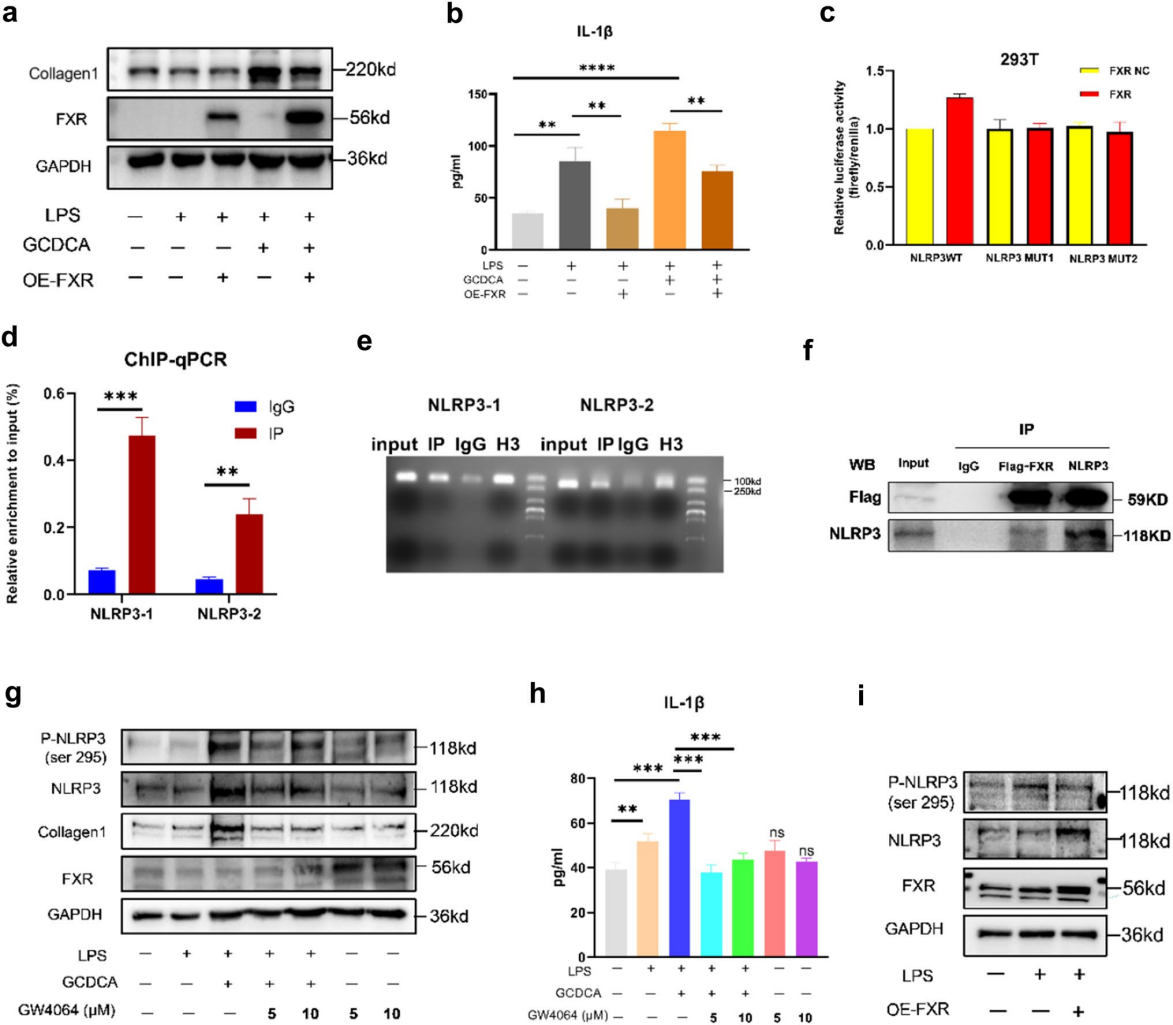
FXR attenuates liver fibrosis by inhibiting the phosphorylation of NLRP3. **a** Collagen 1 expression in cell lysates and IL-1β concentration (**b**) in culture supernatant following FXR overexpression in LX2 cells. Dual-luciferase reporter assays (**c**), ChIP qPCR (**d**), gels map (**e**) and COIP (**f**) of FXR and NLRP3. **g** Western blots of NLRP3, P-NLR3 (ser 295), collagen 1, and FXR in lysates and IL-1β concentration (**h**) in culture supernatant following GW4064 treatment in LX2 cells. **i** Western blots of NLRP3 and P-NLRP3 (ser 295) following FXR overexpression in LX2 cells. *p < 0.05; **p < 0.01; ***p < 0.001, ns: no significance (compared with control). Error bars indicate SEM. Date are from 3 independent experiments

We treated LPS-primed LX2 cells with GCDCA for 48 h based on FXR overexpression. The results showed decreased expression of collagen 1 and IL-1β secretion following overexpression of FXR (Fig. 5a, b). What is the mechanism by which FXR attenuates hepatic fibrosis? To determine whether FXR, as a transcription factor, inhibits the expression of NLRP3 by inhibiting the transcription of NLRP3, we performed dual luciferase reporter (Fig. 5c) and ChIP‒qPCR experiments (Fig. 5d, e), which showed that FXR promotes the transcription of NLRP3 by binding to its promoter. Therefore, to determine whether FXR affects NLRP3 activity at the protein level, we performed a COIP experiment (Fig. 5f) and showed that FXR and NLRP3 bind to each other at the protein level. NLRP3 phosphorylation is an essential initiating factor for inflammasome activation [19, 20]. To test whether FXR affects NLRP3 phosphorylation, we stimulated LX2 with the FXR agonist GW4064 and found a decrease in NLPR3 phosphorylation after FXR activation by GW4064, and IL-1β secretion was also reduced (Fig. 5g, h). NLRP3 phosphorylation level was also reduced after overexpression of FXR (Fig. 5i).

### FXR agonist GW4064 weakens the hepatic fibrosis induced by GCDCA in mice

**Fig. 6:**
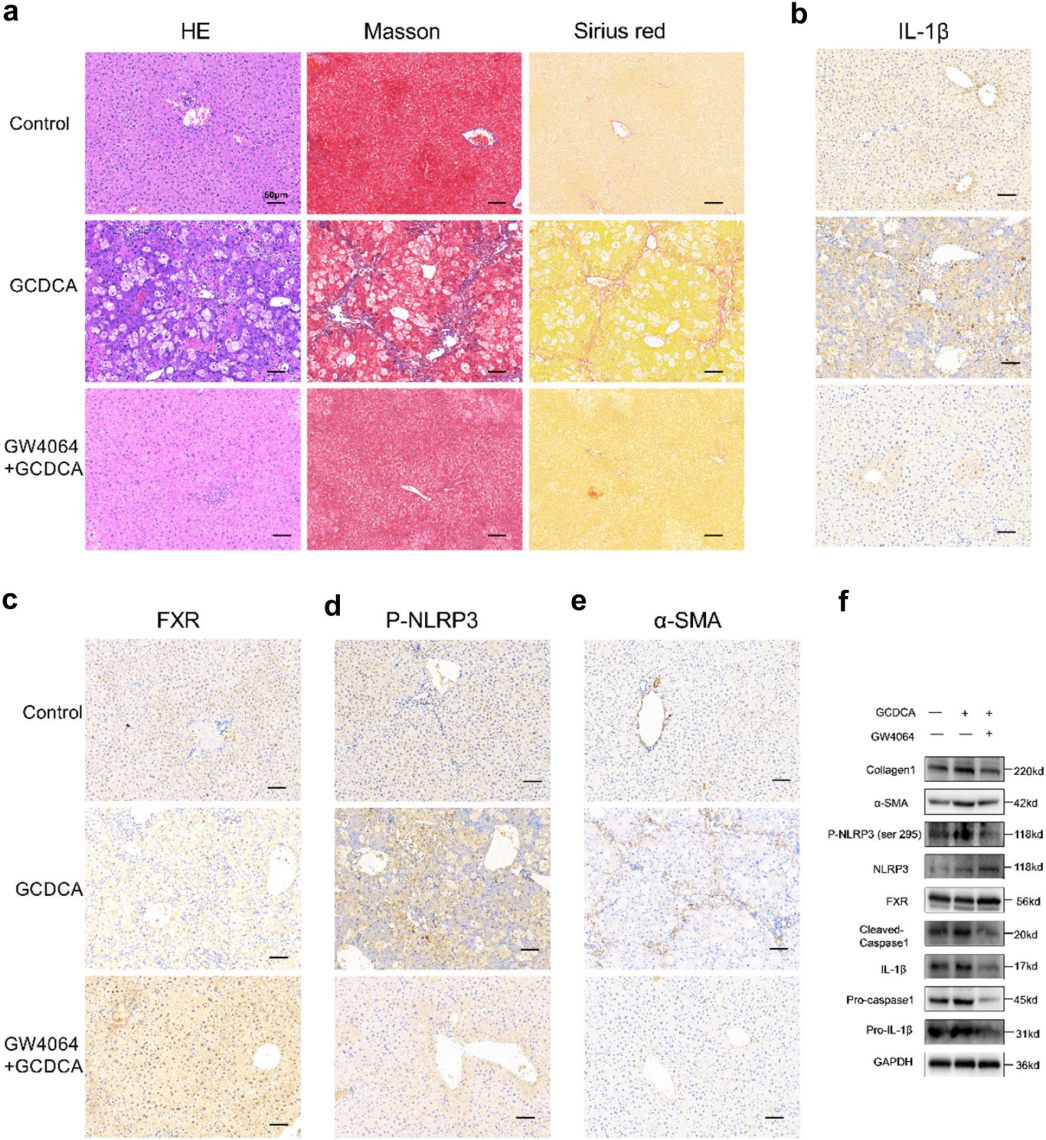
FXR agonist GW4064 weakens the hepatic fibrosis induced by GCDCA in mice. **a** HE, Masson and Sirius red staining of mouse livers. Scale bar: 50 μm. Immunohistochemistry of IL-1β (**b**), FXR (**c**), P-NLRP3 (**d**) and α-SMA (**e**) in mouse livers. Scale bar: 50 μm. **f** The expressions of NLRP3 inflammasome and fibrosis related proteins, P-NLRP3 (ser 295), and FXR were detected by western blotting

To investigate whether FXR activation may affect the occurrence of hepatic fibrosis in vivo, we interposed mice with GW4064 and GCDCA at the same time. HE, Masson’s trichrome, and Sirius Red staining showed that GW4064 decreased GCDCA-induced fibrosis and collagen formation in mouse livers (Fig. 6a). Futhermore, immunohistochemistry of the livers showed significantly higher positivity of FXR and lower positivity of IL-1β, P-NLRP3 and α-SMA in the GW4064 + GCDCA group than in the GCDCA group (Fig. 6b-e). Notably, with the activation of FXR, the protein expressions of Phosphorylated NLRP3 (ser 295), cleaved caspase-1, mature IL-1β (17kd) and fibrosis markers (collagen I and α-SMA) decreased compared to GCDCA group (Fig. 6f).

## Discussion

In liver fibrosis cases, BAs metabolism is disrupted. In the current study, GCA, GCDCA, TCDCA and TCA were significantly increased in patients with liver fibrosis, which was in agreement with other studies [18, 21]. These BAs may be potential indicators in the diagnosis of hepatic fibrosis, especially GCDCA.

Inflammation is critically involved in the initiation and progression of hepatic fibrosis. Patients with liver fibrosis showed higher IL-1β expression in the liver, indicating high levels of liver inflammation in patients with hepatic fibrosis. We hypothesized that hepatic inflammation was caused by elevated bile acids. In vitro, GCDCA induced the activation of LX2 by promoting the expression of the NLRP3 inflammasome and the expression of collagen protein. To demonstrate the role of the NLRP3 inflammasome in activating liver fibrosis, we infected LX2 cells with lentivirus with NLRP3 knockdown and showed that GCDCA-induced liver fibrosis markers were significantly reduced after shNLRP3.

There are researches about BAs induced hepatic inflammation and fibrosis, for example, Guan et al. reviewed that bile acids can cause chronic inflammatory response in cardiometabolic diseases. However, it did not elucidate whether GCDCA could cause liver fibrosis and the mechanism of BAs in the formation of liver fibrosis [22]. Another study observed that feeding mice with GCDCA (0.3%, w/w) and CA (0.1%, w/w) at the same time could lead to hepatic fibrosis, but in the subsequent in vitro study, the authors intervened with CDCA and concluded that bile acids activated hepatic stellate cells through EGFR and MEK 1/2 signaling pathways [23]. There is little research about GCDCA induced liver fibrosis lonely in vivo. Holtmann et al. found that LCA or DCA activate the NLRP3 inflammasome, promoting inflammation and liver injury in mice as well as the activation of HSCs [10]. Li et al. showed that NLRP3 inflammasome activation was the main pathway in liver fibrosis induced by aldosterone [24]. To further determine whether elevated GCDCA can cause liver fibrosis in vivo and the role of NLRP3 in the progression of liver fibrosis, we used GCDCA gavage and CCl_4_ intraperitoneal injection intervention, and the results showed that both of them could induce liver fibrosis in mice. However, few fibrosis events were observed in the NLRP3^−/−^ + CCl_4_ and NLRP3^−/−^ + GCDCA groups, suggesting that the NLRP3 inflammasome pathway may be critically involved in the process of liver fibrosis driven by a diverse array of factors. Our study showed that GCDCA induced the activation of LX2 cells and fibrosis in mice by NLRP3 inflammasome.

In patients with HBV-associated acute liver failure, hepatic transcript levels of FXR were decreased and the levels of NLRP3 were increased compared with normal [15]. In the ER stress induced by tunicamycin, the expression of NLRP3 inflammasome related proteins increased, while the expression of FXR decreased in mouse liver or hepatocytes [15]. In various mouse models of liver injuries, including cholestasis induced by bile duct ligation, acute liver injury induced by intraperitoneal injection of CCl_4_, nonalcoholic steatohepatitis induced by high-fat high-cholesterol diet or methionine and choline-deficient diet, all the protein levels of FXR in the liver decreased [25]. They are in line with our study, with activation of the NLRP3 inflammasome, FXR expression was reduced in LX2 cells and in the liver of mice with CCl_4_-induced and GCDCA-induced hepatic fibrosis compared to the control mice (Figs. 2a, c, 3e, 6f). FXR, activated by GW4064, can reverse the increase of NLRP3 inflammasome caused by ER stress. This mechanism may be that FXR inhibits PERK-CHOP-NLRP3 pathway in hepatocytes induced by ER stress [15]. In another report, the mechanism of FXR negatively regulating the NLRP3 inflammasome was through FXR interacting with NLRP3, as well as caspase 1 in protein levels during sepsis associated with cholestasis [11]. FXR activation not only inhibited the LPS-induced upregulation of NLRP3 and IL-1β in monocytes isolated from the livers of MDR2^−/−^ mice, but suppressed their expression induced by TCDCA in bone marrow–derived macrophages isolated from the WT mouse [26]. Our study showed that the overexpression of FXR could inhibit the secretion of IL-1β and the expression of fibrotic protein collagen1, and then we further investigated the mechanism. When analyzing transcription factors and promoters, we did observe that FXR as a transcription factor can promote the expression of NLRP3 promoter according to the dual-luciferase reporter and ChIP assays. And studies have shown that FXR acts as a transcription activator to initiate downstream transcription process [27, 28]. It is interesting to note that some studies have shown that FXR can affect proteins function by affecting their phosphorylation status [29, 30]. Moreover, the phosphorylation of serine 295 of NLRP3 is necessary for its activation [20]. Therefore, we further analyzed the effect of FXR on the phosphorylation of NLRP3 (ser 295), and the results showed that FXR and NLRP3 were mutually bound in the COIP assay and the activation or overexpression of FXR did inhibit the phosphorylation of NLRP3, thus inhibiting its activity. In vivo, GW4064, an agonist of FXR, could inhibit the inflammation and fibrosis of liver induced by GCDCA in mice, and concomitantly inhibit the increased expression of phosphorylated NLRP3 in GCDCA interference mice.

## Conclusions

In conclusion, our research showed that liver fibrosis can cause a disorder of BA metabolism, and the dysfunctional bile acid can cause inflammation and activate HSCs through NLRP3 inflammasome, thus further aggravating liver fibrosis. Activation of FXR or NLRP3 knockout or knockdown can reduce liver fibrosis.

## Supplementary Information

Below is the link to the electronic supplementary material.

Supplementary file1 (DOCX 252 KB)

## Data Availability

The experimental data sets generated and/or analyzed during the current study are available from the corresponding author upon reasonable request.
