# Correction: Bile acids induce liver fibrosis through the NLRP3 inflammasome pathway and the mechanism of FXR inhibition of NLRP3 activation

**DOI:** 10.1007/s12072-025-10791-w

**Published:** 2025-03-27

**Authors:** Shu Feng, Xingming Xie, Jianchao Li, Xu Xu, Chaochun Chen, Gaoliang Zou, Guoyuan Lin, Tao Huang, Ruihan Hu, Tao Ran, Lu Han, Qingxiu Zhang, Yuanqingxiao Li, Xueke Zhao

**Affiliations:** 1 Department of Infectious Diseases, Affiliated Hospital of Guizhou Medical University, Guizhou Medical University, No. 9 Beijing Road, Guiyang, 550004 Guizhou China; 2 Department of Hepatobiliary Surgery, The First Affiliated Hospital of Guangzhou Medical University, Guangzhou, 510120 Guangdong Province China; 3 Laboratory of Hepatology, Affiliated Hospital of Guizhou Medical University, Guizhou Medical University, Guiyang, 550004 Guizhou China; 4 Department of Hepatobiliary Surgery, Affiliated Hospital of Guizhou Medical University, Guizhou Medical University, Guiyang, 550004 Guizhou China; 5 Department of Cardiovascular Medicine, Guiqian International General Hospital, Guiyang, 550018 Guizhou China

**Correction: Hepatology International (2024) 18:1040–1052** 10.1007/s12072-023-10610-0

In this article, in Fig. 4 part figure b (4b) appeared incorrectly and has now been corrected in the original publication. For completeness and transparency, the incorrect and correct versions are displayed below.

Incorrect Fig. 4:

**Fig. 4:**
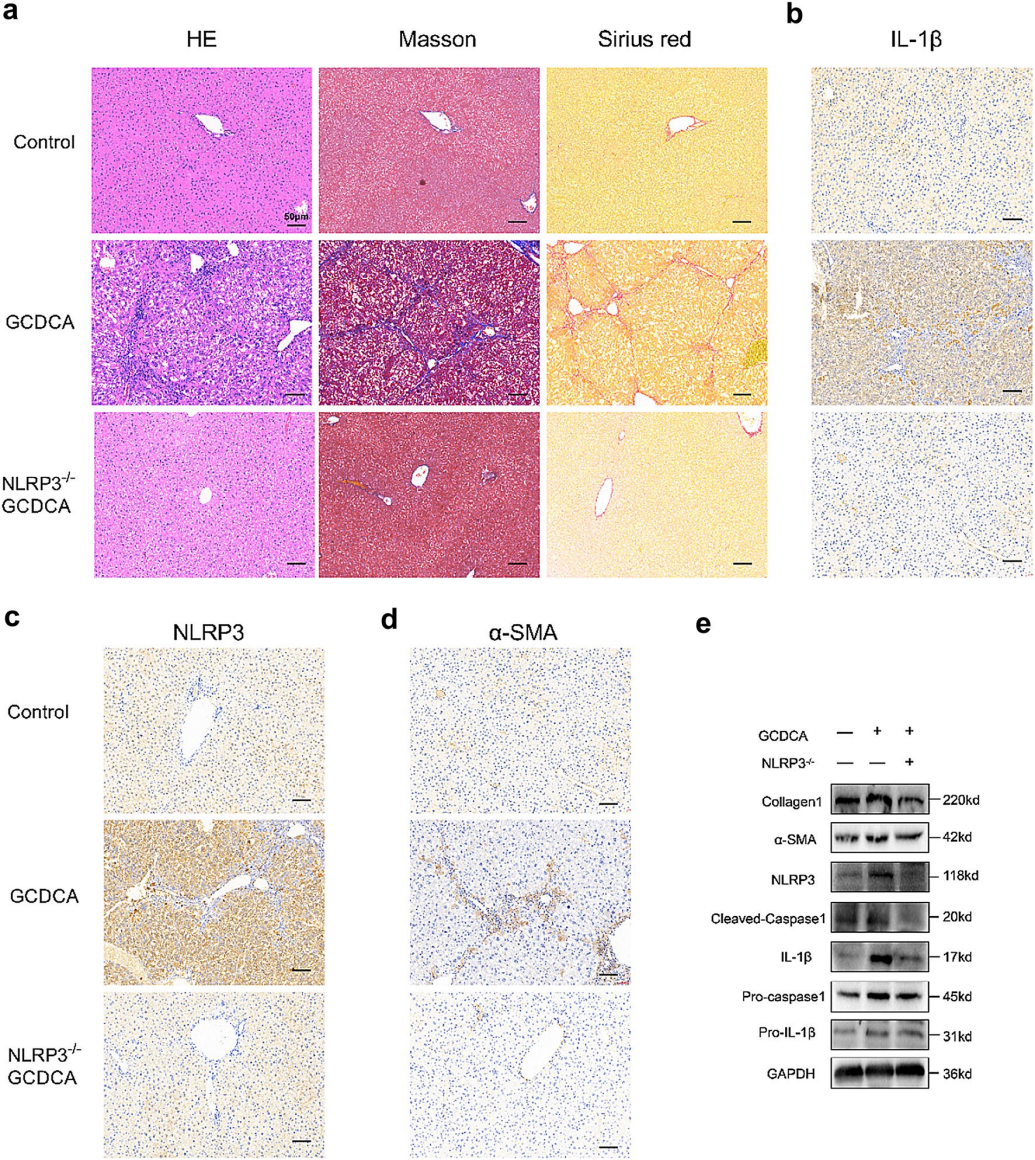
NLRP3 knockout reduces GCDCA-induced liver fibrosis in mice. a HE, Masson and Sirius red staining of mouse livers. Scale bar: 50 μm. b, c, d Immunohistochemistry of IL-1β, NLRP3and α-SMA in mouse livers. Scale bar: 50 μm. e Western blots of NLRP3-relted proteins and fibro-related proteins among groups

Correct Fig. 4:

**Fig. 4:**
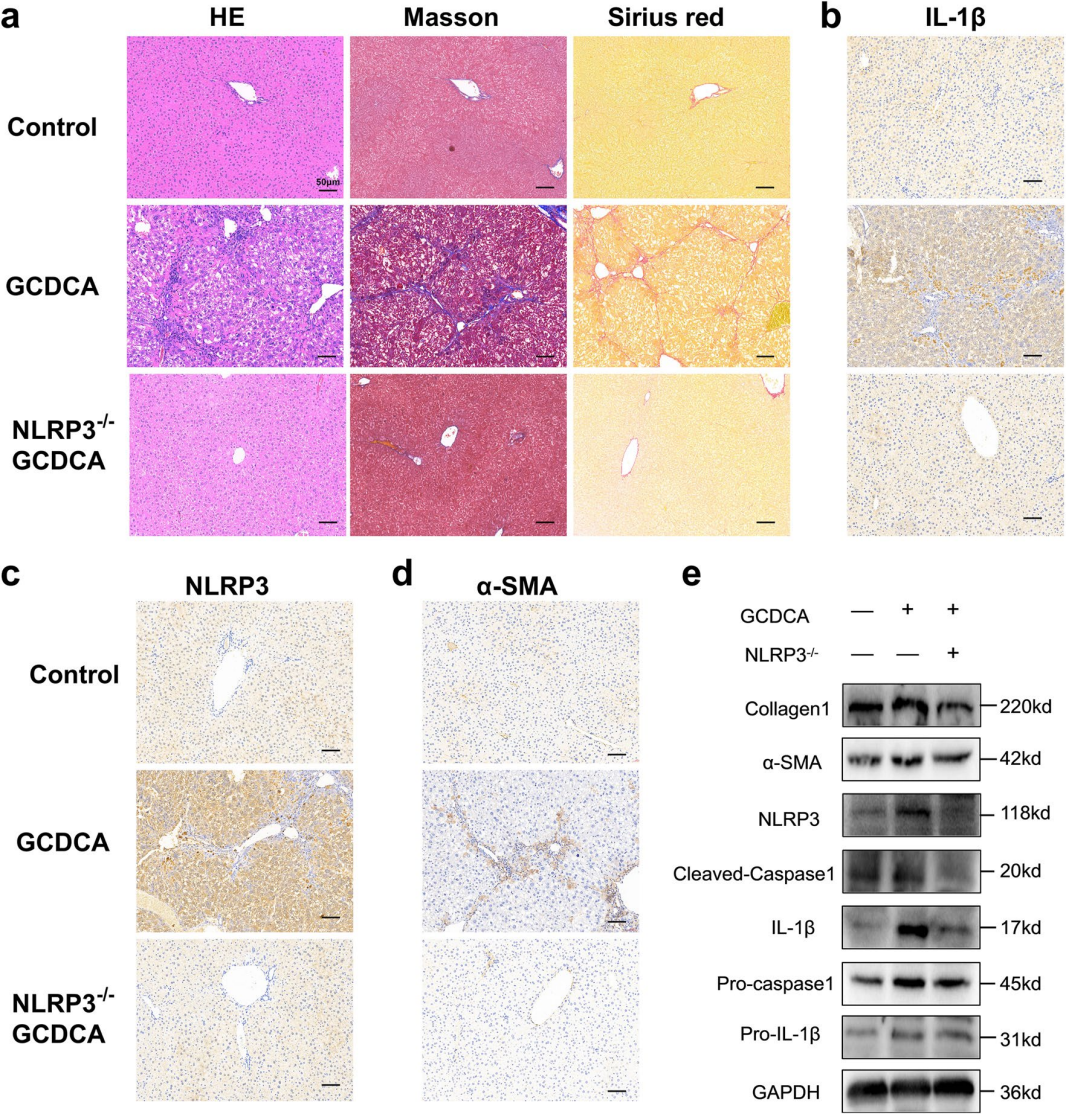
NLRP3 knockout reduces GCDCA-induced liver fibrosis in mice. a HE, Masson and Sirius red staining of mouse livers. Scale bar: 50 μm. b, c, d Immunohistochemistry of IL-1β, NLRP3and α-SMA in mouse livers. Scale bar: 50 μm. e Western blots of NLRP3-relted proteins and fibro-related proteins among groups

The original article has been corrected.

