# Supplementary material for: Bile acids induce liver fibrosis through the NLRP3 inflammasome pathway and the mechanism of FXR inhibition of NLRP3 activation
Source: Hepatol Int. 2024 Jan 3;18(3):1040–52. doi: 10.1007/s12072-023-10610-0 (PMC11126483; doi:10.1007/s12072-023-10610-0)
Supplement: Supplementary file 1 — Supplementary file1 (DOCX 252 KB) [file 12072_2023_10610_MOESM1_ESM.docx]

**Supplemental materials**


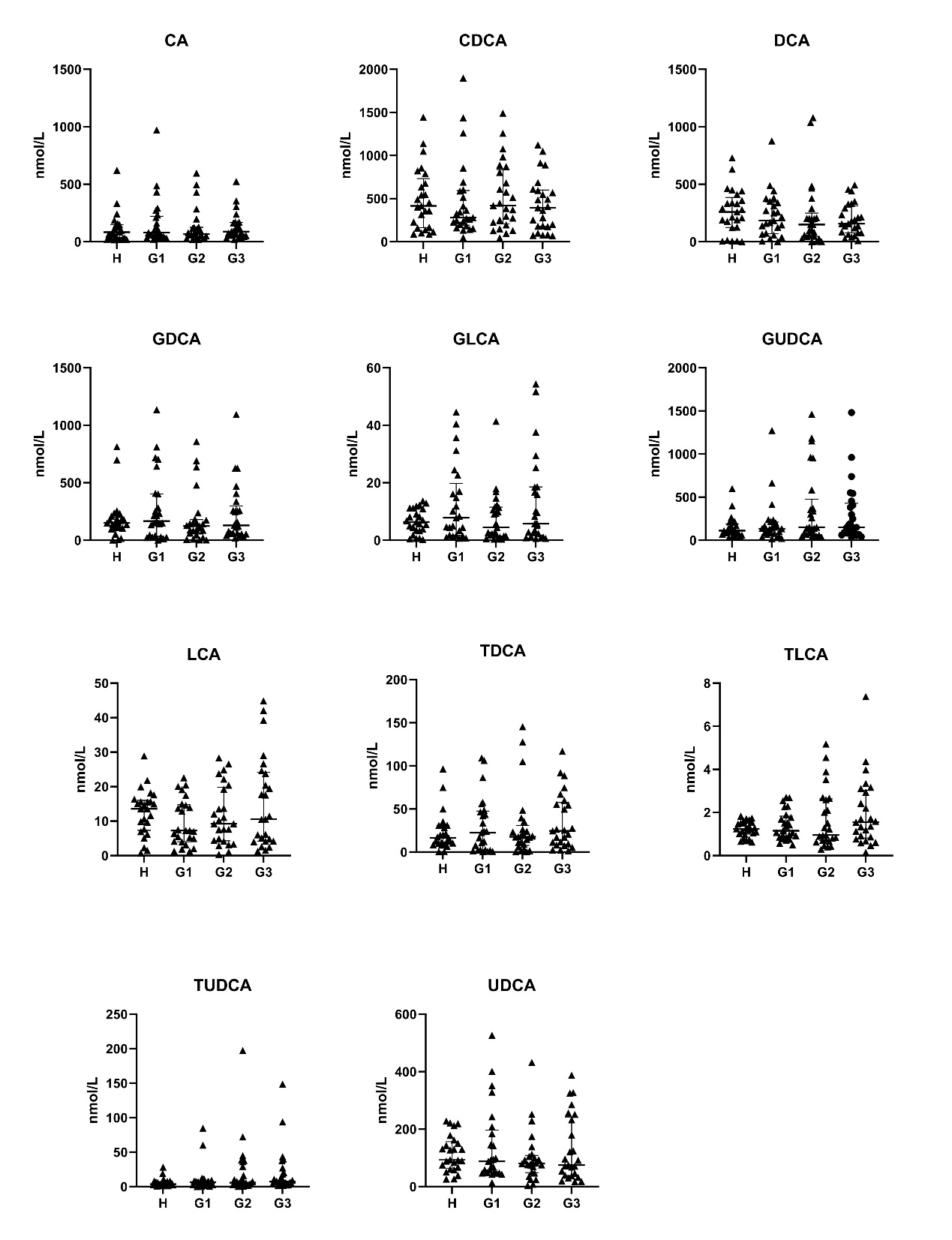


**Figure S1:** The concentration of plasma BAs in various groups. P>0.05 between groups.
